# Practical Notes on Diagnosis and Treatment

**Published:** 1907-12-28

**Authors:** 


### Practical Notes on Diagnosis and Treatment.

**Gonorrhœal Vaginitis.**

In uncomplicated gonorrhœal vaginitis Mr. J. W. Taylor advises a vaginal suppository of silver nitrate (gr. $\frac{1}{4}$) every night, and a douche of crude acetic acid ($\frac{1}{2}$ ounce to 1 pint of water) in the course of the day.

**Phlebitis and Thrombosis.**

When affecting the lower limb the limb should be raised on a pillow, and a mixture of equal parts of lead lotion and laudanum may be applied to relieve the pain. Afterwards careful bandaging should be adopted.

**Antipyrin in Pertussis.**

Dr. F. C. Crandall has found this useful. The drug is best administered with syrup of tolu. The initial dose for a child of one year is $1\frac{1}{2}$ grains every four hours, the intervals being gradually reduced to two hours.

**To Abort a Cold.**

After blowing the nose vigorously rinse out each nostril with a weak (pale pink) solution of potassium permanganate. With the head held well back try to retain some of the solution by plugging each nostril with cotton wool.

**Uræmic Paralysis.**

While the occurrence of convulsions due to uræmia is a common experience, the existence of uræmic paralysis is much less frequent. Hemiplegia is the most common form, and it may be complete; on the other hand, the paralysis may involve only a single limb, or may be restricted to one side of the face.

**Paralysis Agitans.**

Hyoscine is recommended by Dr. R. T. Williamson to lesson the tremors and the restlessness of paralysis agitans. He prescribes gr. $\frac{1}{4}$ in 6 ounces of chloroform water; and of this mixture give two teaspoonfuls, equivalent to $\frac{1}{96}$ grain of hyoscine hydrobromide, for a dose. The dose may be gradually increased to $\frac{1}{75}$ grain. After a time the good effect of the treatment lessens, but if, after an interval, it is renewed, good results again follow.

**Œdema in Trichinosis.**

Trichinosis is one of the rare causes of œdema. In its most characteristic form the œdema occurs in the eyelids and over the eyebrows, and when this appears early in the disease its diagnostic value is considerable. In the hands and feet œdema usually occurs late in the disease. Œdema with slight erythema over the swollen tender muscles is very suggestive.

**Treatment in Tabes Dorsalis.**

The cases which seem to benefit most from a thorough course of mercury and potassium iodide are those in which the interval between the onset of the symptoms of tabes and the syphilitic infection is short, that is not more than five years; those in which there is other evidence of active syphilis; and those cases of early tabes characterised especially by pain in the back and tenderness on percussion of the spine —a symptom which would seem to suggest a subacute or chronic spinal meningitis.—*Dr. Aldren Turner.*

**Tertiary Syphilis.**

In many cases medicinal treatment is only successful when the patient is put on a generous diet. If this fails the treatment should be continued at the seaside.

**Cheyne-Stokes Respiration.**

Occasionally Cheyne-Stokes respiration persists up to the moment of death, but it generally disappears, and the breathing becomes regular for some hours before death.

**The Open-Air Treatment.**

If the patient can only be persuaded to be out of doors, lying, sitting or walking, as the case may be, a large part of each day, and, when indoors, to allow the frest access of fresh air day and night, results may be obtained in almost any district, which will compare satisfactorily with those of the most favoured resorts.—*Dr. R. W. Philip.*

**Pilocarpine in Alopecia.**

Dr. Pringle reports the case of a patient who entirely lost the hair of his scalp, and in whom vigorous local treament had altogether failed. Nitrate of pilocarpine, $\frac{1}{6}$ grain, was then injected into the scalp hypodermically. In a week there was a growth of downy hair over the scalp. The dose was increased to $\frac{1}{3}$ grain with satisfactory results.

**Psoriasis.**

Mr. Hutchinson's favourite local treatment is Acidi Chrysophanic Hydrarg. Ammon, of each grs. x., Liq. Carbonis Detergens, ℳx, Adeps Benzoat. ℥j. Remove all scales, as far as possible, by washing or a warm bath, and then rub the ointment into each patch for half an hour at bedtime. The ointment may be left on all night, but if this is disagreeable it may be wiped (not washed) off.

**Infant Feeding.**

There is a prevalent notion that it is bad for an infant to be fed partly on the breast and partly by artificial means; hence it is suggested that if there is partial failure of the breast milk the child ought to be weaned. This is a mistake, as exact observations have shown that with "mixed feeding" infants thrive better than those fed artificially, even though with the latter all possible care is taken.

**Sodium Chlorate in Dyspepsia.**

This drug has been strongly recommended in dyspepsia, and especially in hyperchlorhydria and gastric ulcer. It should be given two or three times daily, freely diluted and in warm solution. The time should be midway between meals, so as to secure the action of the remedy on the mucous membrane. Not more than two drachms should be given in the twenty-four hours. Large doses may cause hæmoglobinuria.

**Physiological Albuminuria.**

Posture, or the assumption of the erect position, has more to do with the so-called physiological albuminuria than has either diet or bathing. Thus the urine passed before rising in the morning is free from albumen, while afterwards, sometimes in a few minutes, albumen is present. It usually persists until the late afternoon, when commonly it diminishes or disappears.

